# Strategies to Assess Risk for Hereditary Cancer in Primary Care Clinics: A Cluster Randomized Clinical Trial

**DOI:** 10.1001/jamanetworkopen.2025.0185

**Published:** 2025-03-07

**Authors:** Elizabeth M. Swisher, Heather M. Harris, Sarah Knerr, Tesla N. Theoryn, Barbara M. Norquist, Jeannine Brant, Brian H. Shirts, Faith Beers, DaLaina Cameron, Emerson J. Dusic, Laurie A. Riemann, Beth Devine, Michael L. Raff, Rabindra Kadel, Howard J. Cabral, Catharine Wang

**Affiliations:** 1 Division of Gynecologic Oncology, Department of Obstetrics and Gynecology, University of Washington, Seattle; 2 Department of Bioethics and Humanities, School of Medicine, University of Washington, Seattle; 3 Department of Health Systems and Population Health, School of Public Health, University of Washington, Seattle; 4 Institute for Public Health Genetics, School of Public Health, University of Washington, Seattle; 5 Department of Bioethics and Humanities, School of Medicine, University of Washington, Seattle; 6 Collaborative Science and Innovation, Billings Clinic, Billings, Montana; 7 Clinical Science & Innovation Department, City of Hope, Duarte, California; 8 Department of Laboratory Medicine and Pathology, School of Medicine, University of Washington, Seattle; 9 The Comparative Health Outcomes, Policy, and Economics (CHOICE) Institute, University of Washington, Seattle; 10 Department of Pharmacy, School of Medicine, University of Washington, Seattle; 11 Medical Genetics, Mary Bridge Children’s, MultiCare Health System, Tacoma, Washington; 12 Biostatistics and Epidemiology Data Analytics Center (BEDAC), Boston University School of Public Health, Boston, Massachusetts; 13 Department of Biostatistics, Boston University School of Public Health, Boston, Massachusetts; 14 Department of Community Health Sciences, Boston University School of Public Health, Boston, Massachusetts

## Abstract

**Importance:**

Best practices for improving access to assessment of hereditary cancer risk in primary care are lacking.

**Objective:**

To compare 2 population-based engagement strategies for identifying primary care patients with a family or personal history of cancer and offering eligible individuals genetic testing for cancer susceptibility.

**Design, Setting, and Participants:**

The EDGE (Early Detection of Genetic Risk) clinical trial cluster-randomized 12 clinics from 2 health care systems in Montana, Wyoming, and Washington state to 1 of 2 engagement approaches for assessment of hereditary cancer risk in primary care. The study population included 95 623 English-speaking patients at least 25 years old with a primary care visit during the recruitment window between April 1, 2021, and March 31, 2022.

**Intervention:**

The intervention comprised 2 risk assessment engagement approaches: (1) point of care (POC), conducted by staff immediately preceding clinical appointments, and (2) direct patient engagement (DPE), where letter and email outreach facilitated at-home completion. Patients who completed risk assessment and met prespecified criteria were offered genetic testing via a home-delivered saliva testing kit at no cost.

**Main Outcomes and Measures:**

Primary outcomes were the proportion of patients with a visit who (1) completed the risk assessment and (2) completed genetic testing. Logistic regression models were used to compare the POC and DPE approaches, allowing for overdispersion and including clinic as a design factor. An intention-to-treat analysis was used to evaluate primary outcomes.

**Results:**

Over a 12-month window, 95 623 patients had a primary care visit across the 12 clinics. Those who completed the risk assessment (n = 13 705) were predominately female (64.7%) and aged between 65 and 84 years (39.6%). The POC approach resulted in a higher proportion of patients completing risk assessment than the DPE approach (19.1% vs 8.7%; adjusted odds ratio [AOR], 2.68; 95% CI, 1.72-4.17; *P* < .001) but a similar proportion completing testing (1.5% vs 1.6%; AOR, 0.96; 95% CI, 0.64-1.46; *P* = .86). Among those eligible for testing, POC test completion was approximately half of that for the DPE approach (24.7% vs 44.7%; AOR, 0.49; 95% CI, 0.37-0.64; *P* < .001). The proportion of tested patients identified with an actionable pathogenic variant was significantly lower for the POC approach than the DPE approach (3.8% vs 6.6%; AOR, 0.61; 95% CI, 0.44-0.85; *P* = .003).

**Conclusions and Relevance:**

In this cluster randomized clinical trial of risk assessment delivery, POC engagement resulted in a higher rate of assessment of hereditary cancer risk than the DPE approach but a similar rate of genetic testing completion. Using a combination of engagement strategies may be the optimal approach for greater reach and impact.

**Trial Registration:**

ClinicalTrials.gov Identifier: NCT04746794

## Key Points

**Question:**

What is the most effective way to improve assessment of hereditary cancer risk and offer genetic testing in primary care clinics?

**Findings:**

In this 12-month cluster randomized clinical trial of 12 clinics from 2 health care systems with 95 623 patients seen, contact with patients at the time of clinic appointments (point of care) resulted in a significantly higher proportion completing risk assessment compared with email or mail outreach (direct patient engagement). However, the proportion completing testing across the 2 approaches was similar.

**Meaning:**

In this study, the point-of-care approach screened more patients for hereditary cancer risk, but lower testing completion suggests additional efforts are needed to maximize uptake.

## Teaser

This cluster randomized clinical trial compares 2 population-based engagement strategies for identifying primary care patients with a hereditary risk of cancer and offering eligible individuals genetic testing for cancer susceptibility.

## Introduction

Up to 10% of cancers are caused by inherited susceptibility that can be readily identified by commercially available multigene tests.^1^ By identifying genetic susceptibility, targeted risk reduction can reduce the mortality of several hereditary cancers, including breast and ovarian cancer and those associated with Lynch syndrome.^2,3,4,5,6^ Most genetic testing for cancer susceptibility occurs after a cancer diagnosis, which misses an important opportunity for prevention.^1,2,3,4^

Leading professional organizations, including the US Preventive Health Service Task Force and the National Comprehensive Cancer Network (NCCN), have provided recommendations for identifying individuals at risk by using familial and personal histories and offering genetic testing.^7,8^ As the broadest and most prevention-oriented US health care platform, primary care is an ideal context in which to identify hereditary cancer risk prior to a cancer diagnosis. However, family cancer history is infrequently assessed among primary care patients.^9,10^ Evidence is lacking on how to best conduct population-level risk assessment without increasing physician and system burden. Most prior research studies implementing assessment of hereditary cancer risk have been conducted in specialty and/or academic practices or public health settings and evaluated a limited fraction of patients.^11,12,13,14^

We sought to implement and evaluate universal assessment of hereditary cancer risk followed by at-home genetic testing for eligible individuals in 2 community health care systems. We compared 2 novel population-level engagement strategies for risk assessment designed to minimize clinician and health care system burden, using a cluster randomized clinical trial design.^15^

## Methods

### Study Design

The study protocol was approved by the Human Participants Division of the University of Washington (trial protocol in Supplement 1) and follows the Consolidated Standards of Reporting Trials (CONSORT) 2010 reporting guideline.^15^ The Early Detection of Genetic Risk (EDGE) trial evaluated 2 population-based strategies for engaging patients in assessment of hereditary cancer risk and genetic testing between April 1, 2021, and March 31, 2022. Study randomization was conducted at the clinic level. The clinics within each health care system were paired by size before randomization (eFigure 1 in Supplement 2). A study statistician generated a random number for each clinic, with the higher number in each pairing assigned to the first arm. Informed consent was waived for participation by the University of Washington institutional review board as it was deemed minimal risk. However, patients were given the option to decline the risk assessment and any subsequent study participation. Clinics in the first arm used a point-of-care (POC) approach, which engaged patients at the time of a primary care visit. The second arm used a direct patient engagement (DPE) approach, which involved contacting patients via email and postal mail to complete risk assessment online. Two hypotheses were tested: (1) a greater proportion of DPE patients than POC patients would complete risk assessment, and (2) a greater proportion of POC than DPE patients would complete genetic testing.

### Setting and Participants

The study was conducted at 12 primary care clinics from 2 nonprofit multiclinic health care systems (6 clinics each). MultiCare is an urban health care system located in Washington state. Billings Clinic is a more rural health care system with clinics in Montana and Wyoming. These health care systems have been detailed further in the eMethods in Supplement 2 and prior publications.^15^ Inclusion criteria for study participants were English-speaking individuals aged 25 years or older seen for a primary care well visit during the recruitment window (April 1, 2021, to March 31, 2022). Attempts were made to deduplicate patients (eMethods in Supplement 2).

### Study Interventions

Each POC clinic hired a full-time research assistant to approach patients in person at Billings Clinic sites and over the telephone for MultiCare (eMethods in Supplement 2). Billings Clinic POC patients completed the risk assessment on electronic tablets in the clinic. In contrast, MultiCare POC clinics pivoted to telephone engagement for risk assessment 1 week prior to appointments because of a systemwide conversion to telehealth due to the COVID-19 pandemic. For the DPE clinics, emails and postal mailings inviting patients to complete the risk assessment online were sent quarterly by study staff to all patients seen at the clinic in the prior 3 months.

#### Assessment of Hereditary Cancer Risk 

Participants completed an assessment tool for hereditary cancer risk built for the study using existing guidelines, erring on the side of broader capture of individuals at risk (eMethods in Supplement 2).^16,17^ Individuals identified as eligible, based on personal and family cancer history, were then offered at-home genetic testing (eMethods in Supplement 2).^15^

#### Genetic Testing for Eligible Individuals

All testing was done using Color Health’s Hereditary Cancer Genetic Test, a 29-gene panel.^18^ The study paid for all testing, which included genetic counseling through Color Health. Eligible patients were contacted by study staff via telephone or email to explain the genetic testing process and answer questions. No formal pretest counseling was provided unless requested. Test kits were mailed to interested participants after confirmation of mailing addresses. Participants were required to provide an email address and set up an online account to activate their saliva test kit and receive results. Genetic counseling was provided via telephone for all patients with identified pathogenic variants (PVs) and for others by request (eMethods in Supplement 2).

### Outcomes

The study’s 2 primary outcomes were (1) the proportion of patients with a visit who completed risk assessment and (2) the proportion of patients with a visit who completed at-home genetic testing. An intention-to-treat analysis was used to evaluate primary outcomes using the entire study population as the denominator, regardless of whether they were successfully approached. Secondary outcomes included patient-level factors associated with test completion and the proportion of actionable variants among those who completed risk assessment and were identified as eligible for genetic testing. Actionable variants were those that had risk reduction recommendations in NCCN guidelines.^19,20,21^

### Statistical Analysis

Primary outcomes were evaluated using data collected in aggregate at the clinic level. Secondary outcomes were evaluated using data collected at the patient level, exclusively among those completing the risk assessment (eMethods in Supplement 2). Primary and secondary analyses began by examining the distributional characteristics of all study variables in the form of descriptive statistics that included mean (SD) values and quantiles for continuous variables and frequency counts and percentages for categorical variables.

All statistical analyses were conducted using SAS, version 9.4 (SAS Institute Inc). All *P* values were from 2-sided tests and results were deemed statistically significant at *P* < .05. Additional details are in eMethods in Supplement 2.

#### Primary Outcomes

We performed a series of bivariate logistic regression models with scale adjustment for overdispersion and estimated unadjusted odds ratios (ORs) with 95% CIs and associated *P* values testing for differences in odds between study arms, with the DPE approach as the reference category. The dependent variables for these models were the proportion of patients completing the risk assessment and the proportion completing at-home genetic testing, with the number of eligible patients during the 12-month study period serving as the denominator. In adjusted analyses, we controlled for health care system (Billings Clinic or MultiCare) and clinic size (patient populations of ≥8000 or <8000 per year). Exploratory clinic-level analyses compared the proportion of patients approached, identified as eligible for genetic testing, and who ordered an at-home test kit by study arm.

#### Secondary Outcomes

Patient-level analyses were conducted among those who completed the risk assessment and were identified as eligible for genetic testing. We used multivariable logistic regression models to identify factors associated with test completion, accounting for clinic clustering using generalized estimating equations. Analyses tested for heterogeneity of intervention effects among test-eligible patients by including interaction terms in separate models for study arm with (1) health care system and (2) clinic size.

## Results

### Clinic Characteristics

eTables 1 and 2 in Supplement 2 show the clinical site and demographic characteristics, respectively. Most clinic sites were small (<8000 visits per year) and urban, with predominately White patients. Over a 12-month engagement window, 95 623 patients were seen in the 12 clinics (Figure).

**Figure.**
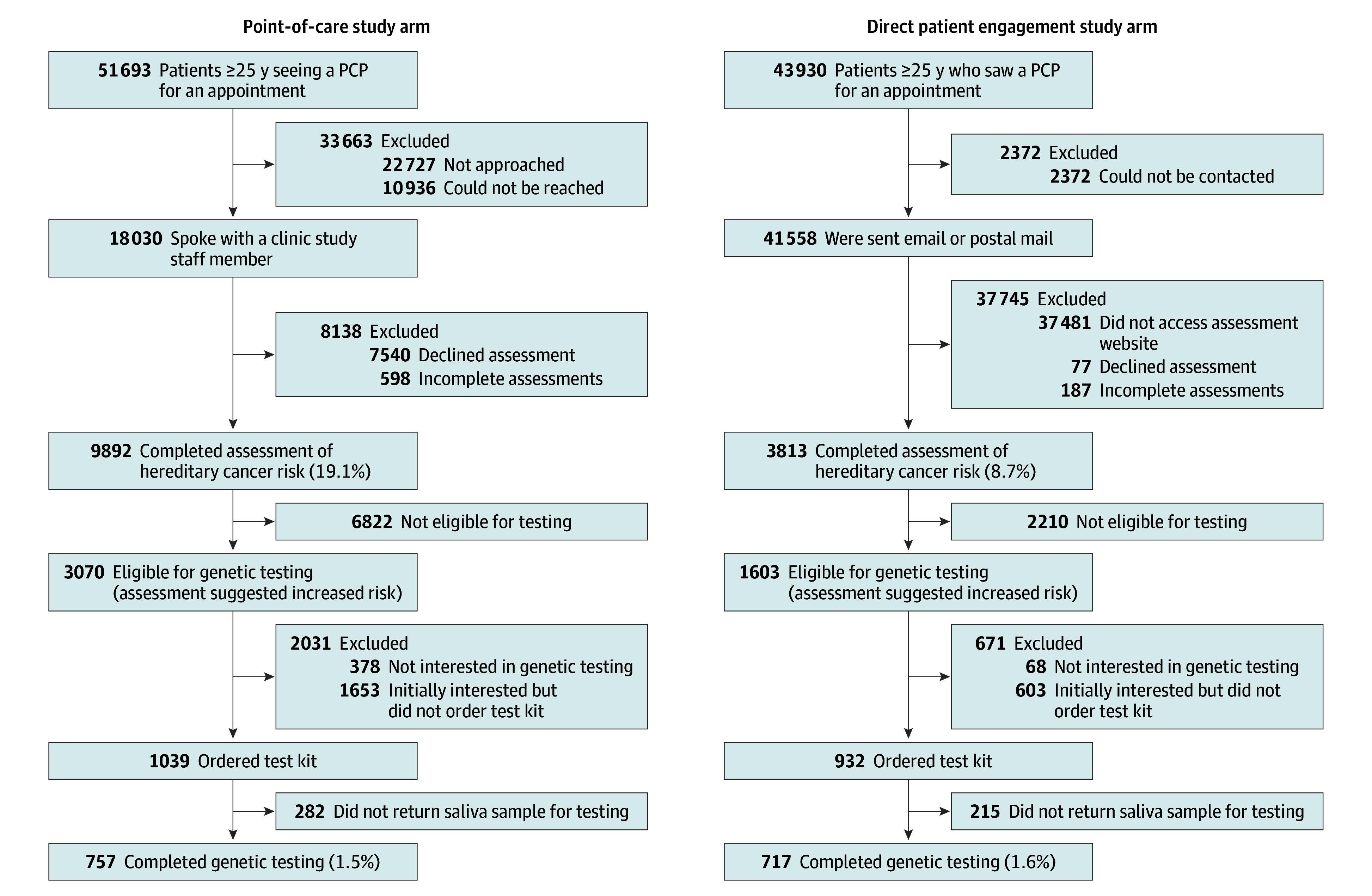
Flow Diagram Flow diagram for the trial showing patient numbers in each arm through the different steps of the study and reasons for dropout at each level. PCP indicates primary care physician.

### Primary Outcomes

Study staff approached 18 030 individuals in the POC arm and sent 41 558 emails or letters in the DPE arm (Table 1). Those who completed the risk assessment (N = 13 705) were predominately female (64.7%) and aged between 65 and 84 years (39.6%). Variability in the proportion of patients approached via POC between clinics was high (range, 20.7%-73.8%) (eTable 3 in Supplement 2). The POC arm had a significantly greater proportion of patients who completed the risk assessment compared with DPE arm (19.1% vs 8.7%) after adjusting for health care system and clinic size (adjusted OR [AOR], 2.68; 95% CI, 1.72-4.17; *P* < .001) (Table 1). However, the proportion of patients completing genetic testing did not differ between the POC and DPE arms (1.5% vs 1.6%; AOR, 0.96; 95% CI, 0.64-1.46; *P* = .86). In exploratory clinic-level analyses, the proportion of patients identified as eligible for testing was significantly higher in the POC arm (5.9% vs 3.6%; AOR, 1.74; 95%CI, 1.23-2.48; *P* = .002). eTable 3 in Supplement 2 provides primary outcomes by clinic site.

**Table 1.** Primary Outcomes

Characteristic	No. (%)			Unadjusted		Adjusted^a^	
	Overall (N = 95 623)	POC approach (n = 51 693)	DPE approach (n = 43 930)	OR (95% CI)	*P* value	OR (95% CI)	*P* value
Patients approached	59 588 (62.3)	18 030 (34.9)	41 558 (94.6)	0.03 (0.01-0.08)	<.001	0.03 (0.01-0.07)	<.001
Assessment completed^b^	13 705 (14.3)	9892 (19.1)	3813 (8.7)	2.49 (1.28-4.85)	.007	2.68 (1.72-4.17)	<.001
Patients eligible for testing and at risk	4673 (4.9)	3070 (5.9)	1603 (3.6)	1.67 (0.99-2.81)	.06	1.74 (1.23-2.48)	.002
Ordered test kit	1971 (2.1)	1039 (2.0)	932 (2.1)	0.95 (0.47- 1.91)	.88	1.02 (0.67-1.54)	.94
Testing completed^b^	1474 (1.5)	757 (1.5)	717 (1.6)	0.89 (0.46-1.76)	.75	0.96 (0.64-1.46)	.86

Abbreviations: DPE, direct patient engagement; OR, odds ratio; POC, point-of-care.

^a^ Adjusted for health care system and clinic size.

^b^ Primary outcomes; the *P* values and 95% CIs were estimated from clinic-level data using logistic regression models with a Pearson scale parameter to account for overdispersion.

### Characteristics of Patients Completing the Risk Assessment

Among participants who completed the risk assessment, those in the POC arm were older than those in the DPE arm (aged 65-84 years, 40.3% vs 37.8%) and less likely to be female (62.3% vs 71.2%), have a personal history of cancer (9.1% vs 10.7%; *P* = .001) or have 2 first-degree relatives with a high-risk cancer (9.5% vs 12.8%; *P* < .001), and, if eligible, order a test kit (33.8% vs 58.1%; *P* < .001) (Table 1 and Table 2). Table 2 outlines clinical and demographic variables of those who completed the risk assessment, while Table 3 provides the factors associated with genetic testing among eligible individuals. Reasons for withdrawal are outlined in the study CONSORT diagram (Figure).

**Table 2.** Clinicodemographic Characteristics of Individuals Completing Assessment of Hereditary Cancer Risk

Characteristic	No. (%)			*P* value
	Overall (N = 13 705)	POC approach (n = 9892)	DPE approach (n = 3813)	
Age, y				
25-44	3053 (22.3)	2233 (22.6)	820 (21.5)	<.001
45-64	4823 (35.2)	3340 (33.8)	1483 (38.9)	
65-84	5427 (39.6)	3986 (40.3)	1441 (37.8)	
≥85	396 (2.9)	329 (3.3)	67 (1.8)	
Not reported	6 (0.04)	4 (0.04)	2 (0.05)	
Sex				
Male	4768 (34.8)	3672 (37.1)	1096 (28.7)	<.001
Female	8872 (64.7)	6159 (62.3)	2713 (71.2)	
Not reported	65 (0.5)	61 (0.6)	4 (0.1)	
Personal cancer history^a^				
No	12 397 (90.5)	8990 (90.9)	3407 (89.4)	<.001
Yes	1308 (9.5)	902 (9.1)	406 (10.7)	
≥2 FDRs with cancer^b^				
No	12 283 (89.6)	8957 (90.6)	3326 (87.2)	<.001
Yes	1422 (10.4)	935 (9.5)	487 (12.8)	

Abbreviations: DPE, direct patient engagement; FDR, first-degree relative; POC, point-of-care.

^a^ Personal history of any high-risk cancer (breast, colon or rectal, endometrial, kidney or urinary tract, melanoma [later dropped], ovarian, pancreatic, prostate, small intestine, and stomach).

^b^ Defined as 2 or more FDRs with a history of any type of cancer.

**Table 3.** Factors Associated With Genetic Testing (of 4673 Patients Assessed as Eligible for Testing)^a^

Characteristic	All sites		
	No.	OR (95% CI)	Adjusted OR (95% CI)
Age, y			
25-44	865	1 [Reference]	1 [Reference]
45-64	1660	0.89 (0.81-0.99)	0.88 (0.79-0.97)
65-84	2002	0.89 (0.77-1.04)	0.93 (0.82-1.06)
≥85	144	0.26 (0.16-0.43)	0.25 (0.14-0.46)
Sex			
Male	1276	1 [Reference]	1 [Reference]
Female	3383	1.13 (0.98-1.30)	1.14 (0.99-1.32)
Personal cancer history^b^			
No	3594	1 [Reference]	1 [Reference]
Yes	1079	0.68 (0.57-0.82)	0.70 (0.60-0.83)
≥2 FDRs with cancer^c^			
No	3470	1 [Reference]	1 [Reference]
Yes	1203	1.20 (1.08-1.33)	1.22 (1.10-1.36)
Study arm			
POC	3070	0.46 (0.32-0.66)	0.49 (0.37-0.64)
DPE	1603	1 [Reference]	1 [Reference]
Health care system			
Billings Clinic	2008	1 [Reference]	1 [Reference]
MultiCare	2665	1.47 (0.88-2.46)	1.56 (1.17-2.08)
Clinic size			
Small	2461	1 [Reference]	1 [Reference]
Large	2212	1.01 (0.52-1.95)	0.98 (0.72-1.34)

Abbreviations: DPE, direct patient engagement; FDR, first-degree relative; OR, odds ratio; POC, point-of-care.

^a^ Tests for interaction examining genetic testing uptake among eligible: Arm × health care system interaction: genetic testing (individual variables include age, sex, personal cancer history, family history of cancer, study arm, size, health care system, and arm × health care system) (MultiCare vs Billings Clinic: OR, 1.20; 95% CI, 0.95-1.52; *P* = .06 for interaction). Arm × clinic size interaction: genetic testing (individual variables include age, sex, personal cancer history, family history of cancer, study arm, health care system, clinic size, and arm × size) (large vs small: OR, 1.27; 95% CI, 0.96-1.67; *P* = .09 for interaction).

^b^ Personal history of any high-risk cancer (breast, colon or rectal, endometrial, kidney or urinary tract, melanoma [later dropped], ovarian, pancreatic, prostate, small intestine, and stomach).

^c^ Defined as 2 or more FDRs with a history of any type of cancer.

### Secondary Outcomes

Among test-eligible participants, the overall test completion rate was 31.5%; the proportion completing genetic testing was significantly lower in the POC arm compared with the DPE arm (24.7% vs 44.7%; AOR, 0.49; 95% CI, 0.37-0.64; *P* < .001). One-fourth of individuals (25.2%) who ordered the test kit never returned it. Other significant factors associated with test completion among test-eligible patients included younger age and 2 or more first-degree relatives with cancer (Table 3). However, test-eligible patients with a personal history of cancer were less likely to complete testing than those without a cancer history.

Genetic testing completion among test-eligible patients was significantly greater in the MultiCare (urban) health care system but did not vary significantly by clinic size (Table 3). In exploratory analyses, there was no statistically significant interaction between study arm and health care system, but a higher rate of genetic testing completion was found among test-eligible patients in the DPE arm relative to the POC arm was more pronounced in Billings Clinic compared with MultiCare (a difference of 26.6% vs a difference of 14.1%; *P* = .06) (eFigure 2 in Supplement 2). Similarly, there was no statistically significant interaction between study arm and clinic size (difference, 15.0% for small clinics vs difference, 25.6% for large clinics; *P* = .09).

Of the 1474 patients who underwent genetic testing, PVs were identified in 123 individuals, including 76 actionable PVs (5.2%), of which 17 were in *BRCA1* or *BRCA2* and 11 were in Lynch syndrome–associated genes (eTable 4 in Supplement 2). Of those without a PV, 35 of 1351 (2.6%) met with the Color Health genetic counselor, including 24 of 1102 (2.2%) with negative results and 11 of 249 (4.4%) with a variant of uncertain significance. Having a personal history of cancer was associated with higher odds of identifying an actionable PV (AOR, 2.16; 95% CI, 1.36-3.44; *P* = .001). The odds of identifying an actionable PV were lower for patients in the POC arm vs patients in the DPE arm who completed testing (3.8% vs 6.6%; AOR, 0.61; 95% CI, 0.44-0.85; *P* = .003).

## Discussion

To our knowledge, the EDGE trial is the first randomized cluster trial comparing approaches to conducting a population-based assessment of hereditary cancer risk in a primary care setting. A systematic review by Guan et al^11^ identified 16 studies between 2005 and 2019 that examined strategies to increase the reach of a genetic risk assessment outside of an oncology or genetic specialty clinic. Only 2 of these studies were randomized clinical trials that compared more than 1 risk assessment approach, and both were conducted in public health settings with small denominators of approached patients.^12,13^ Prior studies in primary care settings have focused mainly on introducing risk assessment or decision support tools for clinicians or for a small subset of patients.^22,23^

Our POC approach resulted in a significantly higher proportion of patients completing the risk assessment, which was contrary to our first hypothesis that we would screen a higher proportion of patients through DPE. The DPE approach involved contacting patients via email or postal mail, whereas the POC approach involved a personal exchange with patients at the time of a primary care visit. Although the proportion of patients approached was lower for the POC arm, the personal engagement provided likely contributed to the significantly higher proportion of patients in the POC arm who completed the assessment of hereditary cancer risk.

The proportion of patients who completed genetic testing was similar between arms, which was contrary to our second hypothesis that the POC approach would result in a higher proportion completing genetic testing. Relative to patients in the POC arm, those in the DPE arm who completed screening were more likely to have a personal history of cancer and 2 or more first-degree relatives with cancer, resulting in a higher proportion who were eligible for testing. Among test-eligible individuals, those in the DPE arm had almost double the test completion rate. Thus, patients in the DPE arm who completed screening were significantly more likely to be eligible for testing and had higher follow-through to complete testing, suggesting the differential selection of patients by arm that should be explored in future studies.

Our 2 engagement approaches were designed to minimize burden on the health care system while implementing a population-based assessment of hereditary cancer risk. The DPE arm used fewer resources than the POC arm, as the POC approach required staff to encourage risk assessment completion for patients with scheduled appointments. Because the proportion of patients completing genetic testing was similar between arms, the DPE approach seems superior. However, because patients in the DPE arm had stronger personal and family cancer histories and different rates of testing completion, these approaches may have reached different populations. A sequential combined strategy starting with a DPE approach followed by a POC approach to assess those missed by the DPE approach might be the optimal strategy for future research efforts to evaluate.

We randomized clinics in 2 different health care systems, 1 urban and 1 more rural, to assess the generalizability of our approaches. Neither system was previously offering routine assessment of hereditary cancer risk. Preexisting differences in the 2 health care systems were further accentuated by the COVID-19 pandemic, which began just prior to study launch. Our urban system adopted telehealth, while the rural system did not. Therefore, the POC clinics differed in implementation, with the more rural system using our previously planned in-person approach and the urban system reaching out to patients via telephone 1 week prior to their appointments. Completion of POC screening was higher in our urban health care system, although variability in the proportion of patients approached via POC between clinics was high (range, 20.7%-73.8%) (eTable 3 in Supplement 2). Our data highlight how implementation may be influenced by contextual factors, both between and within health care systems.^24,25^

Testing completion was also significantly greater among eligible patients from our urban health care system, and exploratory analyses showed this difference was entirely driven by the POC arm (Table 3). There are several likely explanations, including system use of different POC approaches (ie, telephone vs in person). Moreover, the requirement to create an online account with an external company to order testing may also have driven system differences in test completion, as patients from the more rural health care system may have found these additional steps more burdensome.^26^ Some insight may be gained from randomized clinical trials testing strategies to increase uptake of other preventive health services.^23,27,28^ For example, efforts to increase uptake of health interventions (such as opt-out strategies) have been more successful when paired with “effort reduction.”^29^ Eliminating the need to create an online account might reduce effort and increase testing follow-through. Other unmeasured differences in patient populations by system, including genetic health literacy, privacy concerns, differences in health care priorities, or health care distrust^30,31,32^ (perhaps exacerbated by the COVID-19 pandemic), may also have influenced completion of testing disproportionately in the POC arm.

Currently, there are no existing quality metrics for population-based genetic service delivery in primary care with which to compare our rates of risk assessment and genetic test completion. A large observational study using a digital health technology platform across 27 clinics (including 3 primary care clinics) found a 16% test completion rate among those identified as elevated risk and a range of 6% to 53% test completion across the 27 clinics.^33^ Our clinic-level risk assessment rates ranged from 8.7% to 19.1% of age-eligible patients and test completion rates ranged from 24.7% to 44.7% of test-eligible patients, which compares favorably but still leaves substantial room for improvement. We sought to use engagement approaches that would not place additional burden on primary care physicians. However, we may need to partner earlier with physicians to increase testing uptake among eligible patients, as has been necessary with other preventive measures, such as vaccines.^34,35,36^

Actionable PVs were found in 5.2% of tested individuals, higher in the DPE arm than the POC arm (6.6% vs 3.8%), consistent with the more frequent personal history of cancer and the higher rate of 2 first-degree relatives with cancer observed among participants in the DPE arm who completed screening. The frequency of actionable PVs is in line with other large studies of cancer genetic testing among moderate-risk individuals^37^ and higher than that seen in unselected populations (1.5%-2.1%).^38^ Population genetic testing has been proposed as a more efficient and equitable way to identify actionable inherited cancer susceptibility and is being piloted in specific populations.^39,40,41^ Our mean test completion rate of 31.5% of eligible individuals suggests that there may be additional barriers to testing, despite providing home saliva testing kits at no cost. One-fourth of individuals (25.2%) who ordered the testing kit never returned it, despite multiple reminders. This rate is similar to another large trial of at-home testing with different patient selection.^37^ Qualitative analyses are ongoing to determine reasons for testing noncompletion.

### Limitations

This study has some limitations. It was a practical trial during a global pandemic, which created variations in clinic priorities and staffing. Nevertheless, these fluctuations are consistent with system stressors that regularly occur in clinical community health care settings. The most obvious effect of the pandemic on study design was the different POC outreach approach deployed by each health care system, which impedes comparing results in this arm by health care system. For the DPE arm, we were not able to discern who opened the invitation email. Furthermore, patients in the POC arm were approached before appointments and patients in the DPE were approached at variable time points, potentially affecting outcomes. In addition, we did not have individual-level data for patients who did not complete the hereditary risk assessment. The limited number of Hispanic participants and participants from other racial and ethnic minority groups, the requirements for English-language capability, and the need for an email address to complete genetic testing reduce the generalizability of our findings. Finally, provision of genetic testing at no cost may have influenced testing completion rates.

## Conclusions

This cluster randomized clinical trial found that, relative to DPE through email or postal mail, POC engagement with primary care patients led to a greater proportion completing the assessment of hereditary cancer risk with higher investment in health care staffing but a similar proportion completing cancer genetic testing. Using a combination of engagement strategies may be the optimal approach for greater reach and impact.

## Notes

Supplement 1.
Trial Protocol and Statistical Analysis Plan

Supplement 2. **eTable 1. **Clinical Site Characteristics **eTable 2. **Clinical Site Population Demographic Characteristics **eTable 3. **Primary Outcomes Across 12 Clinical Sites **eTable 4. **Pathogenic Variants Identified by Gene and Study Arm **eFigure 1. **Study Design **eFigure 2. **Assessment, Eligibility, and Testing Rates by Study Arm
eMethods.

Supplement 3.
Data Sharing Statement
