# Supplementary material for: Strategies to Assess Risk for Hereditary Cancer in Primary Care Clinics: A Cluster Randomized Clinical Trial
Source: JAMA Netw Open. 2025 Mar 7;8(3):e250185. doi: 10.1001/jamanetworkopen.2025.0185 (PMC11889468; doi:10.1001/jamanetworkopen.2025.0185)
Supplement: Supplement 1. — Trial Protocol and Statistical Analysis Plan [file jamanetwopen-e250185-s001.pdf]

Early **D**etection of **GE**netic risk (EDGE)

Getting genetic testing to the public

NCT04746794

November 16, 2023

Single IRB site of record: University of Washington
Principal Investigator(s): Deborah J. Bowen, Elizabeth M. Swisher

UW IRB ID: STUDY00009476

Date of Initial Approval: 04/02/2020
Date of most recent review and approval: 11/16/2023

Determinations, waivers, and regulations

Requirement	Determination or Waiver
Consent	Waived for randomization (Aim 1) and patients in Aim 3
Documentation of consent	Waived for providers and clinic staff in Aim 3
HIPAA Authorization	Waived for prescreening medical records

Contents

OVERVIEW 4
  Objectives 4
  Study Design 4
  Background 4
PARTICIPANTS 5
  Participants 5
  Inclusion Criteria 5
  Exclusion Criteria 6
  Number of Subjects 6
NON-UW RESEARCH SETTING 6
  Reason for Locations 6
RECRUITING and SCREENING PARTICIPANTS 6
  Recruitment and Screening 6
  Recruitment Materials 7
  Payment to participants 8
PROCEDURES 8
  Study Procedures 8
  Data Collection 11
CONSENT PROCESS 12
PRIVACY AND CONFIDENTIALITY 12
  Privacy protections 12
RISK / BENEFIT ASSESSMENT 12
  Anticipated risks 13
  Data and Safety Monitoring 13
  Anticipated direct benefits to participants 13
  Return of individual research results 13
STATISTICAL ANALYSIS PLAN 14

OVERVIEW

Objectives

The purpose of this study is to determine how to best implement appropriate genetic testing and follow-up care in the primary care setting.

Aim 1: Compare two strategies for identifying members of a primary care clinic's population who have a family or personal history of cancer and encouraging these high-risk individuals to obtain genetic testing for cancer susceptibility mutations. The two methods are: 1) Point of Care (POC) approach, completed in conjunction with a regularly scheduled appointment; and 2) Direct Patient Engagement (DPE), using letters and emails. Individuals will be asked to visit a web site for screening for family/personal history of cancer. Genetic testing will be recommended for those determined to be high risk. Outcomes will be the fraction of the clinic population that participates in the family/personal screening and the fraction of the clinic population that undergoes genetic testing.
Hypothesis 1: DPE screening will result in a higher proportion of active patients who screen for familial cancer risk compared with POC screening.
Hypothesis 2: Of screened patients, POC patients will produce a higher proportion of tested patients compared with DPE.

Aim 2: Identify changes, problems, and inefficiencies in clinical flow and interactions during and after the implementation of genetic testing.

Aim 3: Evaluate the effects of the two methods of implementation on patient, provider, and health system leader reports of benefits and harms, satisfaction, and perceived quality of care.

Aim 4: Evaluate the value (cost-effectiveness) and affordability (budget impact) of each method.

Study Design

This is a mixed-methods study with randomization at the clinic level, assessment of an implementation approach, and a longitudinal observational component.

Background

Preventing cancer mortality through primary prevention activities is an understudied and important area of research. One method of preventing cancer mortality is to engage women and men in genetic testing, to better enable risk stratification and subsequent targeted cancer prevention/surveillance. Identifying patients with high genetic risk for breast, ovary, colon, or other cancers has important ramifications for an individual's healthcare and is recommend in multiple guidelines for high-risk patients. However, genetic risk is often not identified until after a cancer diagnosis due to barriers at several levels. National data suggests that relatively little population-based implementation of clinical genetic testing currently occurs. Provider barriers include inadequacies in hereditary risk recognition, challenges in making patient referrals, and availability of genetic professionals to provide in-depth counseling. Patient barriers include limited knowledge of testing, financial burdens, and inadequate access to specialty genetic testing services. Studies to date of the implementation process have been conducted in urban, high resourced facilities, under optimal conditions, often not at the clinic or system level. We propose to compare the processes and outcomes of two methods of offering testing and to evaluate follow-up care, quality of life, and economic considerations. We also propose to follow

more intensively the patients that test positive for mutations to understand reactions to difficult medical genetic information.

PARTICIPANTS

Participants

12 primary care clinics will be recruited for the study. The clinics will be a mix of urban and rural locations that are part of either the Billings Clinic or the MultiCare Health System.

Patients involved will be age 25 or older and considered active patients at a participating clinic.

Providers, clinic staff, and at least one clinic leader from each clinic will also be included. These individuals will be age 18 or older and employed by the clinic or healthcare system.

Inclusion Criteria

Primary care clinics will be selected with the help of representatives from the Billings Clinic and the MultiCare Health System. The clinics must be primary care settings that are geographically distinct (no patient/provider overlap) with at least 100 active patients and a volunteer contact/champion.

Inclusion criteria for Aim 1

Patients included in Aim 1 must be age 25 or older and considered an active patient at one of the participating clinics (i.e., had at least one visit in the past 12 months). They must also be comfortable reading and writing in English.

Half of the clinics will be randomized into the Point of Care (POC) arm. For these clinics, patients will be approached at the time of their visit and invited to complete the risk assessment questionnaire to be included in the study. It is expected that some patients will not have time to complete the assessment, but all patients who meet the criteria above (25 or older, comfortable reading and writing in English; the current appointment qualifies them as an active patient) and could potentially have time to complete the assessment will be approached.

The remaining half of the clinics will be randomized into the Direct Patient Engagement (DPE) arm. For these clinics, all patients who meet the criteria above (25 or older, active patient, comfortable reading and writing in English) will receive a letter and/or email inviting them to complete the risk assessment questionnaire.

Inclusion criteria for Aim 3

Patients included in Aim 3 must be age 25 or older and considered an active patient at one of the participating clinics (i.e., had at least one visit in the past 12 months). They must also be comfortable reading and writing in English. In addition, patients who test positive for one or more mutations for cancer risk will be followed at an increased schedule to determine any harms due to testing, as well as benefits.

Providers, clinic staff, and the clinic leader from each clinic must be employed by the clinic or healthcare system, 18 years of age or older, and comfortable reading and writing in English. When they complete the baseline survey, they will be asked if they would be willing to do a phone interview. A random sample of those who agree to be interviewed will be contacted.

Inclusion Criteria for Aim 4

Providers, clinic staff, and the clinic leader from each clinic must be employed by the clinic or healthcare system, 18 years of age or older, and comfortable reading and writing in English.

Exclusion Criteria

N/A

Number of Subjects

As the clinics participating in the study have not yet been selected and we do not yet know how many patients would be considered "active" at each clinic, we are not able to estimate the total number of individuals who will be in the study.

We will recruit a total of 12 clinics.

We plan to consent approximately 6,000 patients (to complete surveys and interviews).

We plan to consent approximately 250 providers, clinic staff, and clinic leaders.

We estimate that approximately 200,000 patients will have the opportunity to complete the risk assessment questionnaire and around 3,000 will complete the genetic testing.

Of the patients who complete genetic testing, we anticipate that approximately 200 will participate in a previvor assessment and intervention (described below) because they test positive for one or more mutations.

NON-UW RESEARCH SETTING

Reason for Locations

We approached primary care clinics in the Northwest, using existing connections with ITHS. The clinics had to have staff members interested in this research project and be agreeable to the proposed engagement activities. We were also looking for clinics from two different healthcare systems and a mix of urban and rural locations, to make results more widely applicable.

RECRUITING and SCREENING PARTICIPANTS

Recruitment and Screening

Primary care clinics will be identified and approached by representatives of the Billings Clinic and the MultiCare Health System. The clinic will need to have a staff member willing to champion the study and assist with implementation. We will also be looking for a mix of urban and rural locations.

Aim 1

To compare two strategies to identify and test high-risk individuals within primary care clinics, the 12 clinics will be randomized such that half will use the first method and half will use the second.

> Point of Care (POC)
>
> For clinics in the POC arm, patients with a clinic appointment will be approached either in person or over the phone by designated clinic staff (e.g., medical assistant, receptionist). The clinic staff will determine whether a patient should be approached by reviewing their EMR, using the eligibility criteria defined in section 2.2.
>
> Direct to Patient Engagement (DPE)

For clinics in the DPE arm, we plan to reach out to every patient that meets the eligibility criteria defined in section 2.2. This outreach will be done using mail and email communication. Contact information will be pulled from the EMR by the clinic staff or a healthcare representative. Outreach materials will clearly state the role of the clinic in the study and as the source of the patient's contact information.

Aim 3

A random sample of active patients will be selected from each clinic and asked to complete a baseline survey. This sample will come out of the list of patients who meet the eligibility criteria defined in section 2.2. The clinic or a healthcare system representative will pull the contact information for the selected patients from their electronic medical record (EMR). The study staff will reach out using mail and email. Outreach materials will clearly state the role of the clinic in the study and as the source of the patient's contact information (see attached Study Recruitment Templates). This process will be repeated approximately 12 months later, to obtain a second snapshot of the patient population.

We are also interested in interviewing patients who have reached various stages in the risk assessment and genetic testing process. The patient names and contact information will be obtained by clinic staff or directly from the patient through the risk assessment survey.

For previvors (patients who test positive for one or more mutations) we receive the results from Color Genomics from patients who have agreed to allow Color to share their results and we implement the previvor assessment and intervention procedures, using email and telephone.

Providers, clinic staff, and clinic leaders at each clinic will be identified by a representative at the clinic. All of these individuals will be contacted. Contact information will come from the clinic's employee directory or a similar source. Outreach will be conducted using mail and email and will clearly state the role of the clinic in the study and as the source of the individual's contact information (see attached Study Recruitment Templates). A random sample of those who agree to be interviewed will be contacted for an interview.

Aim 4

This aim will include the providers, clinic staff, and clinic leaders at each clinic that were identified in Aim 3. All of these individuals will be contacted. Contact information will come from the clinic's employee directory or a similar source. Outreach will be conducted using email to complete a longitudinal survey. Emails will be collected at the end in order to follow-up.

<u>Recruitment Materials</u>

Individuals invited to participate in the surveys and interviews will receive a letter and/or email introducing the study.

Patients visiting clinics in the POC arm will be given a tablet with the risk assessment website loaded (in-person visits), or walked through the risk assessment over the phone by a clinic staff member. A document with examples of talking points has been uploaded into Zipline. Patients in the POC arm may be approached by clinic staff that they are familiar with from previous visits to the clinic.

Patients in the DPE arm will receive a letter and email (for those who have an email address) introducing the study and providing a link to the study website.

Previvors will receive a phone call from study staff to explain the purpose of the previvor assessment. In this call, study staff will explain the purpose of the call, their role, and a brief overview of the assessment and an invitation to complete an interview in conjunction with the assessment. Study staff will empathize the patient's participation in the assessments and interviews is voluntary. The recruitment email, if patient is not contactable by phone, will have similar language. Both forms of outreach will give the study and a local clinic contact's contact information for patient's reference. If patient wishes to proceed, they will be sent a link to the REDCap survey to fill out the assessment and informed a study staff member will follow up to schedule an interview when they have had a chance to complete the assessment.

Payment to participants

Participants will be compensated for survey and interview time using gift cards provided by the research team. They will receive the gift cards ($50 for providers and clinic leaders in Aim 3, $10 for patient surveys and baseline previvor interviews, $20 for patient follow-up previvor interviews, and $10 for providers, staff and clinic leaders in Aim 4) within a month after each completed interview or survey.

The typical amount a patient would receive is $10 (for completing a single survey). The typical amount a provider would receive is $50 (for completing a single survey). The greatest total amount a participant could receive is $240 (a provider completing the baseline survey and interview, at $50 each, the follow-up survey and interview, at $50 each, and all 4 economic surveys, at $10 each).

Non-monetary compensation

High risk patients will receive genetic testing at no cost to them and those who complete the testing will have access to free genetic counseling. Providers and staff will have access to free CME credits as part of completing training provided by the study.

PROCEDURES

Study Procedures

We propose a clinic-level randomized trial to evaluate two methods of engaging patients in population-based testing for cancer risk.

Baseline Assessment

Before randomizing the clinics, a baseline assessment will be completed.

Clinics: The clinics will be asked to provide aggregate data that will be used to describe their patient population.

Patients: A random sample of patients will be selected from each clinic and asked to complete a baseline survey. This baseline survey will take approximately 20 minutes to complete and will be done online or on paper. Reminders to complete the survey will be sent by email and/or mail.

Providers and clinic staff: Providers and staff at the clinics will be asked to complete a baseline survey online or on paper. A subset will be asked to complete an interview. Interviews will be performed over the phone and will be recorded so that the transcripts can be analyzed.

Randomization of clinics

Once the baseline assessment is complete, randomization will occur at the level of the clinic (within healthcare system). We will block on healthcare system and clinic size to help ensure comparable arms.

Patient Engagement

Arm 1: Point of Care (POC). Patient engagement in the POC arm will involve approaching patients completing routine visits (either in-person or through telemedicine). We will either use electronic tablets in the waiting room or walk the patient through the questions over the phone to screen for familial cancer risk. Patients flagged as high risk based on simple personal or family cancer history will go on to be offered genetic testing (see Testing below).

Arm 2: Direct Patient Engagement (DPE). Patient engagement in the DPE arm will involve contacting patients by mail and email. The outreach will provide a link to the online familial cancer risk screening tool. If flagged as high risk, patients will be offered genetic testing (see Testing below).

Note: The three components below are included to provide additional context and describe the clinical procedures that will be taking place as part of normal, recommended practice. These are not research procedures.

Provider and Staff Engagement

Training will be provided on genetics and guidelines for high risk patients. This training will consist of webinars and online materials and will include Continuing Medical Education (CME) credits, to encourage participation. In addition, Dr. Norquist, an investigator on this study, will be available for consult via phone or email to assist with any questions related to genetic testing and patient care recommendations.

Clinic Engagement

To ensure that patients with abnormal results receive appropriate follow-up care, we will guide participating clinics in creating a system to organize and systemize the follow-up care. While each of the clinics may tailor the workflows to specific clinical needs, the core components of follow-up will be similar across participating clinics including education and support of the providers involved in care; changes to the EMR to cue providers and patients of the needs for care; and up-to-date guidelines support for options such as chemoprevention and changes in screening frequency and/or type.

Testing

Patients who complete the risk assessment questionnaire and are found to be high risk should be offered genetic testing. This recommendation is part of standard clinical guidelines. Patients in the POC arm will be sent an email with more information and will be connected to the Color Genomics website to complete genetic testing, if desired. Patients in the DPE arm will be sent from the risk assessment questionnaire directly to a site with more information and connected to the Color Genomics website to complete genetic testing, if desired. For the genetic testing, a testing kit will be mailed to the patient. Three to four weeks later, results will be sent to the patient and their provider.

Follow-up Assessment

The study will track how many patients complete the risk assessment questionnaire and how many complete genetic testing.

Approximately 12 months after the initial baseline assessments, another random sample of patients and all of the providers and the clinic leader at each clinic will be asked to complete a survey. The survey will take approximately 20 minutes to complete and will be done online or on paper. Reminders to complete the survey will be sent by email and/or mail. A subset of providers will be interviewed. As with the baseline interview, the interviews will be performed over the phone and will be recorded so that the transcripts can be analyzed.

Process Analysis

To obtain feedback on the risk assessment and genetic testing process, and determine what factors play into a patient's decision making along the way, we will interview samples of patients who have reached various stages. One sample will be taken from those patients who are not interested in completing the risk assessment (e.g., select the "skip survey" option in the risk assessment or decline to complete it when approached by clinic staff in the POC clinics). A second sample will be from those who complete the risk assessment. A third will be from patients who are eligible for the genetic testing, but decide not to complete it. And a final sample will be taken from patients who complete the genetic testing process. Interviews will be completed by phone and recorded.

As an additional level of analysis, we will survey participants who ordered a genetic testing kit but did not complete it. The survey will ask about the testing process, their reasons for ordering a kit and why they didn't return it, general thoughts about genetic testing, and a section of demographics (age, sex, education, etc). A paper copy of the survey will be mailed to these participants, in addition to email outreach. The survey may be completed on paper or online.

Previvor Activities

All patients who test positive for one or more mutations will discuss their testing process with a trained interviewer during an interview before they receive results, and at 6 months after they receive results. In addition, we will invite previvors to complete a survey (separate from the baseline assessment that a random sample received) and an interview. These will be done at separate time points from the other follow up assessments as previvors will be identified at varying intervals (as they test). The assessment will be administered at baseline (when they first receiver their results) and at 6 months post-results. The interviews will happen in conjunction with the assessments, at a time the patient and interviewer arrange. For previvors we have uploaded into zipline the previvor report, a document that is meant to help patients understand what their next steps are. And, we have uploaded a document detailing the measures that we will sue with previvors at baseline, before they receive the results, and at 6 months post results receipt.

Aim 4 Activities

Providers, clinic staff, and clinic leaders will be sent a link to an anonymous REDCap survey via email that will ask them about their individual effort and time spent launching and maintaining the study at their respective clinics.

Providers, clinic staff, and clinic leaders will be asked for their email once the survey is completed if they wish to proceed with follow-up. Their emails will be stored with their results once they opt-in for follow-up.

Data Collection

We are asking for general approval of the surveys and interviews that will completed by participants. The topics are described below and more detail can be found in the uploaded EDGE Measures Table document.

The risk assessment questionnaire completed by all active patients asks about their personal cancer history as well as that of any relatives (cancer type, age at diagnosis, relationship to patient). It also asks if any family member has had a mutation in a cancer gene identified. The surveys completed at baseline and throughout the course of the study by samples of patients will provide additional information about the clinic's patient population in regard to demographics, quality of life, satisfaction with care, genetic literacy and self-efficacy, feelings about genetic testing, trust in their provider and the healthcare system, and familial communication. The patient interviews will assess the engagement approach and the patient's reasons for responding (or not responding). We will also ask about the potential benefits and harms associated with each step of the risk assessment and genetic testing process. The provider/staff baseline survey will cover the organization's leadership and readiness for change. Providers will be asked about their background and training, and about their genomic confidence and self-efficacy. The second survey at 12m will reassess provider's genomic confidence and self-efficacy. The provider/staff baseline interview will ask about perspectives on the clinic and healthcare system in regard to risk assessment and genetic services, intervention characteristics (e.g., evidence strength & quality), and the external climate. The interviews at 12m will cover their evaluation of the testing process and clinical activities related to results provision.

The most sensitive questions we will ask (in relation to the previvor assessments) are as follows:

1. Identities of marginalization
    a. Questions about feeling a part of a group or feeling like the participant is getting the resources that they need
2. Level of coercion during process of participation and source
    a. During the testing process did you feel pressure from staff or providers to give a sample for genetic testing?
3. Areas of concern due to positive results (morbidity, mortality, financial stress, discrimination, QOL)
    a. Do you have any concerns about your health now that you have a positive mutation result?
4. Perceived barriers of PGx (pharmacogenomics) in Primary Care
    a. Do you perceived you will have challenges managing your medication and accessing medication?
5. Level of influence from PCP in decision to participate
    a. Did you work with your provider during or after the testing process?
6. Self-rated level of distress from positive result
    a. How do you feel right now? Any changes since you got your mutation findings?

7. Modalities used for self-direct information gathering
    a. Do you have any thoughts about the testing process?
8. Utilization rate of PGx in Primary Care
    a. Will you inquire about prophylactic treatments with your PCP as a result of your results?

CONSENT PROCESS

We have received waivers of consent for clinic randomization and for patients surveyed as part of Aim 3.

We have received a waiver of documentation of consent for providers and clinic staff surveyed and interviewed as a part of Aim 3.

Electronic presentation of consent information
For Aims 3 and 4, participants completing surveys and interviews will be directed to a REDCap survey. The first page of the survey will have a brief overview of the study and will include contact information for the study staff. The recruitment email for Aim 4 contains consent information as well.

For Aim 1, although consent is being waived so as to reduce bias, patients completing the risk assessment questionnaire will be presented with a brief description of the study and the type of information the risk assessment questionnaire will collect, as well as contact information for the study staff. Patients will be given the option to either start the survey or skip the survey and answer some brief questions as to why they do not want to complete the survey.

PRIVACY AND CONFIDENTIALITY

Privacy protections
Outreach materials will clearly identify the clinic that provided the individual’s contact information and emphasize that this a general mailing simply based on them having been seen at a clinic participating in the study. No personal medical information was used to determine if they receive a mailing or not and they are free to opt out of any additional outreach. The consent information for those being interviewed will state that audio recordings of interviews will be transcribed in a manner that removes identifying information and the recordings and transcripts will be destroyed after the records retention period required by state and federal law.

Previvors should not have any privacy concerns. We will state how we have obtained their information in recruitment and all assessments will be administered through REDCap. The consent information for those being interviewed will state that audio recordings of interviews will be transcribed in a manner that removes identifying information and the recordings and transcripts will be destroyed after the records retention period required by state and federal law.

RISK / BENEFIT ASSESSMENT

Anticipated risks

Risks include possible loss of confidentiality and psychological distress when understanding one's personal risk of developing cancer. In other studies, we have seen minimal negative reaction to testing information. However, we will monitor this process and measure any harm that might come to patients as a result of participation in this study.

The risk for the previvors is primarily distress (knowing they have a mutation in a gene associated with cancer). . In other studies, we have seen minimal negative reaction to testing information. Our assessment also try to ascertain this information as well. Previvors cannot be discriminated against due to their mutation status by employers or insurers as they are protected by federal law (Genetic Information Nondiscrimination Act)

We do not anticipate any risk to providers/clinic staff in any of our aims. At minimum, we ask providers and staff to reflect on their workplace and this may bring about negative feelings. The Aim 4 surveys are anonymous to clinic-level research staff (but not UW research staff) so there is no risk to employability or reputation.

Data and Safety Monitoring

We will invite a Data Safety Monitoring Board to review our procedures and data, helping to ensure safety and confidence in the study procedures by participants and scholars. The DSMB will be composed of at least three people, a medical expert, a genetic counselor, and an advocate. They will review the study protocol, the procedures for recruitment and assessment, and the intervention documents to insure adherence to equity and to other ethical principles. They will review data at least annually to note any unanticipated negative effects, and will work with the PIs and investigators to consider changes in the procedures if necessary.

Anticipated direct benefits to participants

Patients found to be at high risk will have access to genetic testing and genetic counseling. The study will cover the cost of these activities.

Return of individual research results

Patients who complete the risk assessment questionnaire will have more information about their potential cancer risk. Those at high risk, for example, may benefit from more frequent screening and would meet the clinical guidelines for genetic testing. Patients that go on to have genetic testing will receive results that may lead a provider to recommend surgery or other interventions to reduce the risk of developing cancer.

Patients who complete the risk assessment questionnaire with a result that is not high risk will receive that information in the same way as patients with a high risk result. The result will be returned to the patient with the caveat that although they are not at high risk based on their family history, there may be environmental and behavioral factors that increase their potential risk of developing cancer. Patients who complete genetic testing with no mutations identified will receive a results report indicating this and explaining that there are other factors that can influence cancer risk, as well as the possibility of genetic links that are not yet known.

Note: the study is helping facilitate genetic testing, but the testing is <u>not</u> a research procedure.

Patients who complete the risk assessment questionnaire will receive their results online after answering the last question. Patients with a high risk result will asked to provide their email address so that the study staff may contact them about proceeding with genetic testing. Patients who are not at high risk will have the option of providing their email address to receive a brief email with some online resources containing more information about cancer risk in general. Patients who complete the genetic testing will receive their results through the Color website three to four weeks after submitting their sample. The testing process involves setting up an online account and the patient will be able to choose whether or not they want to view their results. The results report explains the findings and provides information about how to interpret and use the results. Patients are also given the contact information to speak with a genetic counselor who can further explain the results and possible next steps.

STATISTICAL ANALYSIS PLAN

The primary outcomes are the fraction of the clinic population that participates in the risk assessment screening and the fraction of the clinic population that undergoes genetic testing. We will look at how well the interventions did in terms of getting people to be screened and tested, and how it varies across clinics within treatment arms.

The outcomes are binary, i.e., whether or not each individual in the clinic population completed screening or genetic testing, and individuals are clustered within clinics. Only a minimal amount of identifying data will be required for the screening (ex: age, sex), therefore we plan to analyze two sets of data. The first includes data collected in aggregate at the clinic site level and the second includes data collected at the patient level and integrated additional data at the site level. Analyses of the primary outcomes will use the clinic as the unit of analysis, with the outcome variable for each clinic being the percent of that clinic’s population who complete the screening assessment. We will perform a series of bivariate logistic regression models with scale adjustment for overdispersion. We will also use multivariable logistic regression to estimate and test the relative odds between the study arms for the primary outcomes.

We will estimate the value (cost effectiveness) of one screening strategy over the other, using the incremental cost effectiveness ratio (ICER) to determine which strategy provides more value for the investment made. We will express each of these outcomes as the cost per patient screened and the cost per patient tested.
