# Supplementary material for: Strategies to Assess Risk for Hereditary Cancer in Primary Care Clinics: A Cluster Randomized Clinical Trial
Source: JAMA Netw Open. 2025 Mar 7;8(3):e250185. doi: 10.1001/jamanetworkopen.2025.0185 (PMC11889468; doi:10.1001/jamanetworkopen.2025.0185)
Supplement: Supplement 2. — eTable 1. Clinical Site Characteristics eTable 2. Clinical Site Population Demographic Characteristics eTable 3. Primary Outcomes Across 12 Clinical Sites eTable 4. Pathogenic Variants Identified by Gene and Study Arm eFigure 1. Study Design eFigure 2. Assessment, Eligibility, and Testing Rates by Study Arm eMethods. [file jamanetwopen-e250185-s002.pdf]

Supplementary Online Content

Swisher EM, Harris HM, Knerr S, et al. Strategies to assess risk for hereditary cancer in primary care clinics: a cluster randomized clinical trial. *JAMA Netw Open.* 2025;8(3):e250185. doi:10.1001/jamanetworkopen.2025.0185

eTable 1. Clinical Site Characteristics
eTable 2. Clinical Site Population Demographic Characteristics
eTable 3. Primary Outcomes Across 12 Clinical Sites
eTable 4. Pathogenic Variants Identified by Gene and Study Arm
eFigure 1. Study Design
eFigure 2. Assessment, Eligibility, and Testing Rates by Study Arm
eMethods.

This supplementary material has been provided by the authors to give readers additional information about their work.

© 2025 Swisher EM et al. *JAMA Network Open.*

eTable 1. Clinical Site Characteristics

Clinical Site	Overall	POC	DPE
Healthcare System			
Billings Clinic	6	3	3
MultiCare	6	3	3
Clinic size			
Small (<8000 visits/year)	8	4	4
Large (≥8000 visits/year)	4	2	2
Location (RUCC)[a]			
1 (metro; 1 million+)	6	3	3
3 (metro; <250,000)	3	1	2
7 (nonmetro; <20,000)	3	2	1

[a] Rural-Urban Continuum Codes; definitions available at https://www.ers.usda.gov/data-products/rural-urban-continuum-codes/.

© 2025 Swisher EM et al. *JAMA Network Open.*

eTable 2. Clinical Site Population Demographic Characteristics[a]

Characteristic	No. (%) Overall (n = 94043)	POC (n = 49637)	DPE (n = 44406)
Age			
25 - 44	21878 (23.3)	11729 (23.6)	10149 (22.9)
45 - 64	31880 (33.9)	16372 (33.0)	15508 (34.9)
65 - 84	35136 (37.4)	18908 (38.1)	16228 (36.5)
85+	5149 (5.5)	2628 (5.3)	2521 (5.7)
Sex			
Male	38260 (40.7)	20409 (41.1)	17851 (40.2)
Female	55775 (59.3)	29223 (58.9)	26552 (59.8)
Decline to answer	8 (0.0)	5 (0.0)	3 (0.0)
Race			
AI/AN	1154 (1.2)	506 (1.0)	648 (1.5)
Asian	3171 (3.4)	1279 (2.6)	1892 (4.3)
Black	2344 (2.5)	857 (1.7)	1487 (3.3)
Hawaiian/PI	566 (0.6)	242 (0.5)	324 (0.7)
White	81741 (86.9)	44471 (89.6)	37270 (83.9)
More than one race	1974 (2.1)	845 (1.7)	1129 (2.5)
Unknown / No data	3093 (3.3)	1437 (2.9)	1656 (3.7)
Ethnicity			
Hispanic	1960 (2.1)	934 (1.9)	1026 (2.3)
Non-Hispanic	46864 (49.8)	26538 (53.5)	20326 (45.8)
Unknown / No data	45219 (48.1)	22165 (44.7)	23054 (51.9)
Insurance coverage[b]			
Medicaid	10348 (10.9)	4610 (9.2)	5738 (12.8)
Medicare	39092 (41.2)	20800 (41.5)	18292 (40.9)
Private	47700 (50.3)	25644 (51.2)	22056 (49.3)

Abbreviations: AI/AN, American Indian/Alaska Native; PI, Pacific Islander
[a]Demographic characteristics represent English-speaking patients 25 years of age or older, who had had an appointment at the clinic in 12 months from August 15th, 2021 to August 14th, 2022.
[b]Totals exceed 100% as some patients had more than one type of insurance.

© 2025 Swisher EM et al. *JAMA Network Open.*

eTable 3. Primary Outcomes Across 12 Clinical Sites

MultiCare Health System						
**Site Number**	**1 [a]**	**2 [b]**	**3 [b]**	**4 [a]**	**5 [a]**	**6 [b]**
Patients with a visit	11423	10821	7517	5641	5409	3493
Approached	10714 (93.8%)	2646 (24.5%)	3371 (44.8%)	5227 (92.7%)	5007 (92.6%)	2578 (73.8%)
**Assessment completed**	**1171 (10.3%)**	**1937 (17.9%)**	**2169 (28.9%)**	**699 (12.4%)**	**573 (10.6%)**	**1459 (41.8%)**
Eligible for GT / at risk	474 (4.1%)	570 (5.3%)	630 (8.4%)	313 (5.5%)	225 (4.2%)	453 (13.0%)
Ordered test kit	288 (2.5%)	230 (2.1%)	286 (3.8%)	181 (3.2%)	135 (2.5%)	197 (5.6%)
**Testing completed**	**225 (2.0%)**	**187 (1.7%)**	**192 (2.6%)**	**134 (2.4%)**	**104 (1.9%)**	**145 (4.2%)**

[a]Direct Patient Engagement (DPE) site
[b]Point of Care (POC) site

Billings Clinic Health System						
**Site Number**	**7 [a]**	**8 [b]**	**9 [a]**	**10 [b]**	**11 [b]**	**12 [a]**
Patients with a visit	13196	18397	5011	4701	6764	3250
Approached	12663 (96.0%)	5185 (28.2%)	4697 (93.7%)	975 (20.7%)	3275 (48.4%)	3250 (100.0%)
**Assessment completed**	**944 (7.2%)**	**2347 (12.8%)**	**270 (5.4%)**	**423 (9.0%)**	**1557 (23.0%)**	**156 (4.8%)**
Eligible for GT / at risk	402 (3.0%)	766 (4.2%)	112 (2.2%)	154 (3.3%)	497 (7.3%)	77 (2.4%)
Ordered test kit	232 (1.8%)	134 (0.7%)	59 (1.2%)	58 (1.2%)	134 (2.0%)	37 (1.1%)
**Testing completed**	**183 (1.4%)**	**94 (0.5%)**	**44 (0.9%)**	**47 (1.0%)**	**92 (1.4%)**	**27 (0.8%)**

[a]Direct Patient Engagement (DPE) site
[b]Point of Care (POC) site

© 2025 Swisher EM et al. *JAMA Network Open.*

eTable 4. Pathogenic Variants Identified by Gene and Study Arm

Gene	Overall	POC	DPE
Actionable	76	29	47
APC	6	3	3
ATM	9	1	8
BARD1	2	1	1
BRCA1	7	2	5
BRCA2	10	5	5
BRIP1	1	0	1
CDKN2A	2	1	1
CHEK2	11	4	7
MITF	10	7	3
MLH1	1	0	1
MSH2	1	0	1
MSH6	2	1	1
PALB2	2	1	1
PMS2	7	3	4
RAD51D	5	0	5
Non-Actionable[a]	47	23	24
CHEK2	7	4	3
MUTYH	39	18	21
NBN	1	1	0
**Total**	**123**	**52**	**71**

[a]Pathogenic variants classified as non-actionable included heterozygous PV's in autosomal recessive susceptibility genes (i.e. MYTYH) or low penetrant alleles without clear clinical recommendations from NCCN or other professional organizations (i.e., low penetrant CHEK2 missense variants and NBN).

© 2025 Swisher EM et al. *JAMA Network Open.*

eFigure 1. Study Design

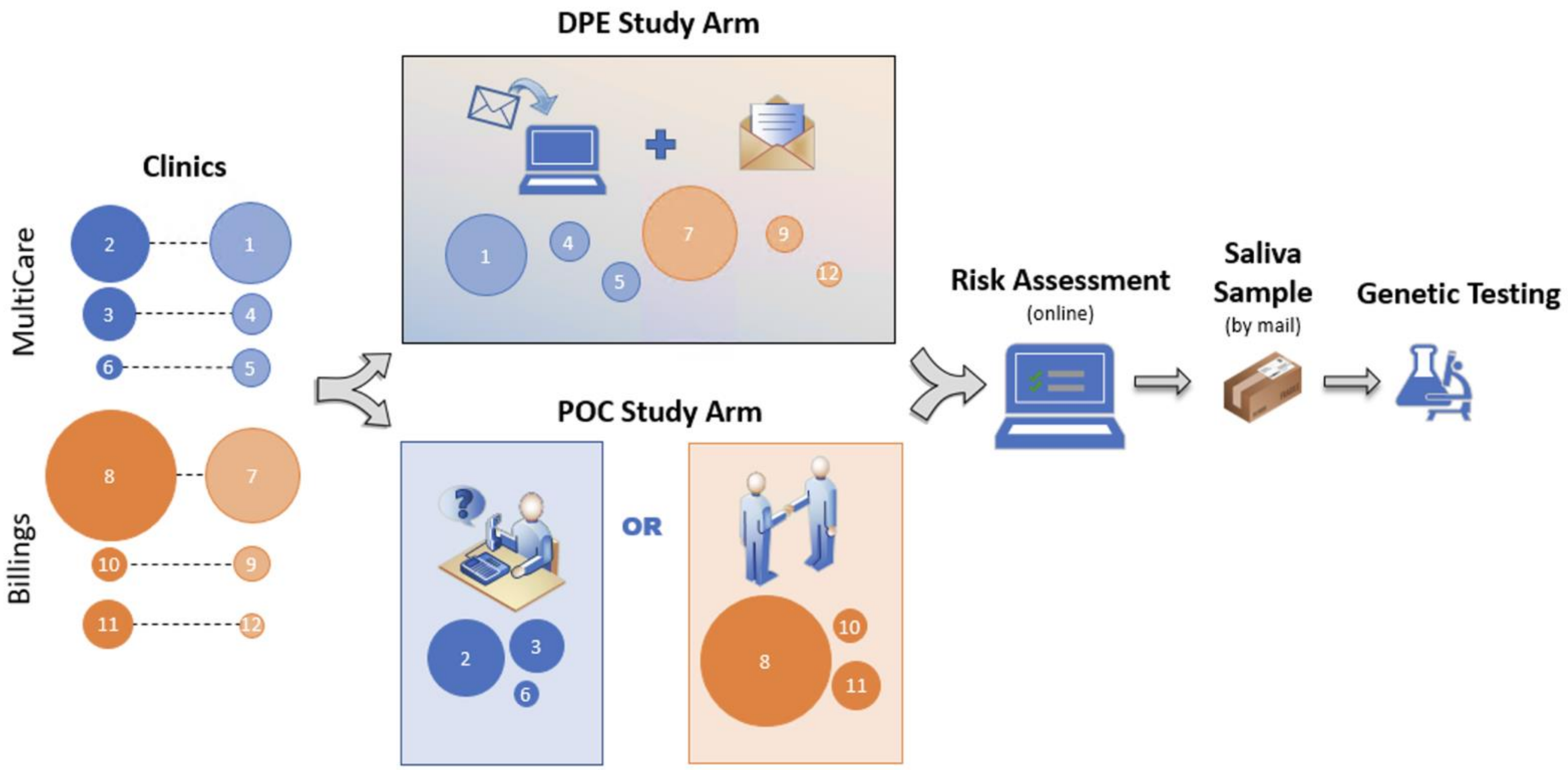

Figure demonstrates randomization into direct-patient-engagement (DPE) and point-of-care (POC) study arms. The colored circles represent the clinics with circle size relative to clinic size and blue circles representing MultiCare clinics and orange circles representing Billings Clinic clinics. Randomization occurred at the clinic level with balance for healthcare system and clinic size. The POC study arm differed by healthcare system with MultiCare using a telephone approach 1 week prior to the clinic appointment, and Billings Clinic using an in-person approach immediately prior to the appointment.

© 2025 Swisher EM et al. *JAMA Network Open.*

eFigure 2. Assessment, Eligibility, and Testing Rates by Study Arm

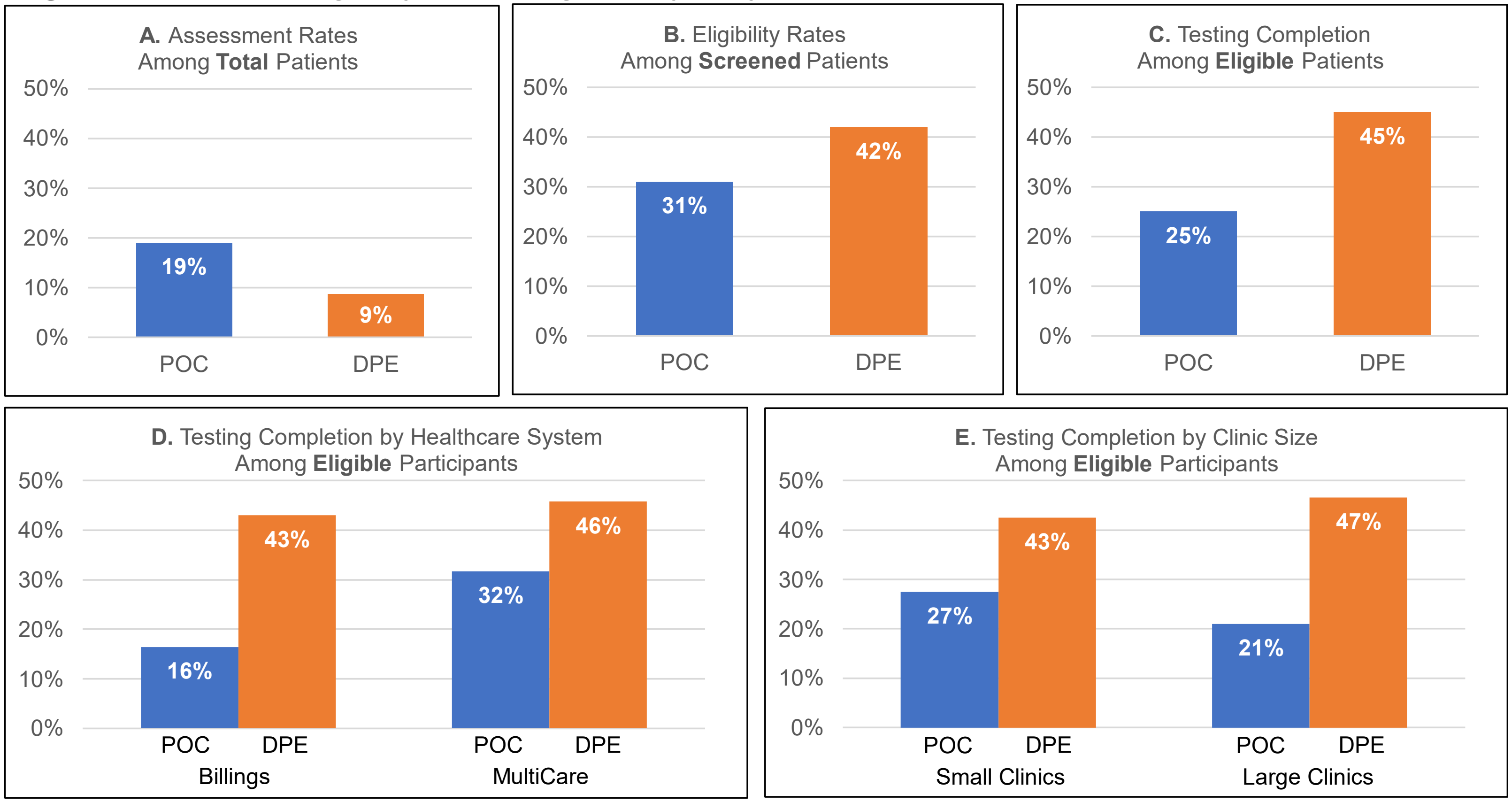

Abbreviation: POC, point-of-care. DPE, direct-patient-engagement.
A. Proportion completing hereditary cancer risk assessment between study arms.
B. Proportion eligible for testing among patients completing hereditary cancer risk assessment by study arm.
C. Proportion completing testing among eligible patients by study arm.
D. Proportion completing testing among eligible patients by study arm and healthcare system. Billings adjusted OR = 0.39 (95% CI: 0.23 – 0.67). MultiCare adjusted OR = 0.55 (95% CI: 0.54 – 0.57).
E. Proportion completing testing among eligible patients by study arm and clinic size. Small clinics adjusted OR = 0.55 (95% CI: 0.47 – 0.65). Large clinics OR = 0.36, (95% CI: 0.21 – 0.61).

© 2025 Swisher EM et al. *JAMA Network Open.*

eMethods.

EDGE Study Hereditary Cancer Risk Assessment: Online Tool

General Demographics

1. What is your age?
2. What is your sex assigned at birth?
3. What is your current gender identity?

Personal Cancer History

4. Have you ever had any of the following type(s) of cancer? (check all that apply)
   - ❑ breast, female
   - ❑ breast, male
   - ❑ colon (large intestine) or rectal
   - ❑ endometrial (uterine)
   - ❑ kidney (renal) or urinary tract
   - ❑ ovarian
   - ❑ pancreatic
   - ❑ prostate
   - ❑ small intestine
   - ❑ stomach (gastric)
   - ❑ None of the above

   For each cancer selected:

   "How old were you when you were first diagnosed with [____] cancer?" - text box for response

   If prostate was selected:

   "Have you had prostate cancer with positive lymph nodes, metastases (spread to another body site), or that was GLEASON 8 or higher?" - radio buttons: yes, no
5. Have you ever had colon polyps removed?
   - ❑ No
   - ❑ Yes

   If yes:

   "How many?" - radio buttons: less than 5, 5-9, 10 or more, unknown

Immediate Family Members' Cancer History

6. Have any of the following relatives ever had cancer?
   - ❑ your mother
   - ❑ your father
   - ❑ your sister
   - ❑ your brother
   - ❑ your child
   - ❑ None of the above
   - ❑ Unknown - no information available

   If sister, brother, and/or child selected, for each relative type selected:

   "How many of your [sisters/brothers/children] have had cancer?" - text box with validation to only accept integers with a minimum of 1 and a maximum of 5

© 2025 Swisher EM et al. *JAMA Network Open.*

For each individual:

"Which of the following type(s) of cancer have they been diagnosed with? (check all that apply)" - List of cancers presented pulled from those in #4 above, with the addition of "unknown cancer type" as an option.

For each instance of female breast cancer, colon, endometrial, kidney, or pancreatic cancer:

"How old were they when they were first diagnosed with [____] cancer?" - radio buttons: 50 or younger; Between 51 and 65; Older than 65; Not sure, not able to guess

Maternal Family Members' Cancer History

7. On your mother's side of the family, have any of the following relatives ever had cancer?

- ❑ your aunt
- ❑ your uncle
- ❑ your grandmother
- ❑ your grandfather
- ❑ None of the above
- ❑ Unknown - no information available

If aunt or uncle selected, for each relative type selected:

"How many of your [aunts/uncles] have had cancer? - text box with validation to only accept integers with a minimum of 1 and a maximum of 5

For each individual:

"Which of the following type(s) of cancer have they been diagnosed with? (check all that apply)" - List of cancers presented pulled from those in #4 above, with the addition of "unknown cancer type" as an option.

For each instance of female breast cancer, colon, endometrial, kidney, or pancreatic cancer:

"How old were they when they were first diagnosed with [____] cancer?" - radio buttons: 50 or younger; Between 51 and 65; Older than 65; Not sure, not able to guess

If any maternal relative had cancer:

"Do you have Ashkenazi Jewish ancestry on your mother's side of the family?" - radio buttons: yes, no, unknown

Paternal Family Members' Cancer History

8. On your father's side of the family, have any of the following relatives ever had cancer?

- ❑ your aunt
- ❑ your uncle
- ❑ your grandmother
- ❑ your grandfather
- ❑ None of the above
- ❑ Unknown - no information available

If aunt or uncle selected, for each relative type selected:

"How many of your [aunts/uncles] have had cancer? - text box with validation to only accept integers with a minimum of 1 and a maximum of 5

For each individual:

© 2025 Swisher EM et al. *JAMA Network Open.*

"Which of the following type(s) of cancer have they been diagnosed with? (check all that apply)" - List of cancers presented pulled from those in #4 above, with the addition of "unknown cancer type" as an option.

For each instance of female breast cancer, colon, endometrial, kidney, or pancreatic cancer:

"How old were they when they were first diagnosed with [____] cancer?" - radio buttons: 50 or younger; Between 51 and 65; Older than 65; Not sure, not able to guess

If any paternal relative had cancer:

"Do you have Ashkenazi Jewish ancestry on your father's side of the family?" - radio buttons: yes, no, unknown

EDGE Study Hereditary Cancer Risk Assessment: Eligibility Criteria for Genetic Testing

One or more of the following factors would make an individual eligible for genetic testing as part of the EDGE Study. Evaluation of responses in the online tool performed in real time, with the final result presented at the end of the assessment.

- Personal cancer history according to ACMG guidelines (i.e. ovarian cancer any age, breast cancer age 50 or less, colorectal cancer <50 year, renal cell <50 years, male breast cancer any age)
- Personal history of 2 or more different cancer types- from the list provided.
- 10 or more colon polyps
- Any first degree relatives with cancer that would trigger genetic testing as in #1.
- Two or more relatives with breast cancer, one age 50 or less.
- Two or more relatives with same type of cancer on one side of the family (excluding prostate cancer over 65).
- Three or more cancers on one side of the family (excluding prostate cancer over 65).
- Two or more first or second degree relatives with ovarian cancer
- One or more first or second degree relatives with ovarian cancer and 1 or more first- or second-degree relatives with breast cancer
- One or more first degree relatives with both ovarian and breast cancer
- Personal history of pancreatic cancer at any age, or any first or second degree relatives with pancreatic cancer diagnosed before age 65
- Colon (or rectal) and endometrial cancer on the same side of the family
- Prostate cancer with positive lymph nodes, metastases (spread to another body site), or that was GLEASON 8 or higher *(only asked as part of personal history)*
- Jewish ancestry (Ashkenazi/Eastern European) and at least one incidence of cancer on the same side of the family *(only asked about ancestry if at least one cancer was reported, excluding prostate cancer over 65)*
- Personal history of one of the listed cancers plus unknown maternal or paternal history

Study Setting

The Billings Clinic and MultiCare Clinic systems were selected with help from the Northwest Participant and Clinical Interactions Network (NCI; https://www.iths.org/community/partners/crn/pci/). MultiCare is the largest not-for-profit urban healthcare system in Washington State with primary care practices, urgent cares, and specialty

© 2025 Swisher EM et al. *JAMA Network Open.*

services including a Genetic Services system. Billings Clinics serve Montana, Wyoming, and the Dakotas. It has a network of federally qualified health centers (FQHCs), primary care services, specialty services, and inpatient care settings. Genetic services are available, but on a limited basis.

Participating clinics had, on average, 13 active providers [3-46 providers] that saw study participants. Billing Clinic had an average of 18 [8-46 providers] active providers per clinic. MultiCare had an average of eight [3-13 providers] active providers per clinic. The study was designed to circumvent the provider to reduce burden, but the risk assessment was offered prior to appointments to allow for follow-up questions for the provider. There was no established study champion or provider requirements.

Study Staff

Each POC clinic hired a full-time research assistant to approach patients. For Billings Clinic, the RA presented the tablet to the patient and offered to help them if needed. For MultiCare, the RA asked the patient questions over the phone and entered the information online on their behalf.

The DPE work was limited to less than a full day's work once every three months (quarterly) rather than the daily full-time coverage at the POC clinics. As such, the DPE clinics did not hire any additional staff. At MultiCare, a project manager pulled the patient lists and uploaded them to the mail provider and to RedCap. At Billings, the research nurse who was part of the study staff pulled the lists and worked with REDCap and the mail provider. The letter sent out to patients inviting them to participate was approved by clinic investigators.

Further communication was facilitated by UW research assistants and staff. Following completion of the risk assessment, UW staff contacted patients and answered questions prior to ordering testing.

Risk Assessment Responses and Patient Follow-up

The risk assessment tool used in the study was built on the REDCap platform hosted at the University of Washington. The risk assessment was refined twice after launch. Melanoma was removed out of concern that it was being confused with skin cancer in general and an "unknown family history" option was added. No information from the screener was uploaded to the patients' medical record or clinic electronic health records (EHRs).

When individuals completed the risk assessment, they received one of the following prompts:

(1) Thank you for taking this risk assessment! Based on your answers, we would like to offer you genetic testing. If you would like to learn more about this opportunity for genetic testing, please provide your email address and telephone number below and we will email you with more information and next steps.
(2) Thank you for taking this risk assessment! Based on your answers, we do not believe genetic testing would be helpful. Most cancers are not genetically linked and generally speaking, most people do not have a significant family history of cancer. This does not guarantee that you will never get cancer. Cancer can be caused by a combination of genetics and other environmental and behavioral factors. It is important that you continue

© 2025 Swisher EM et al. *JAMA Network Open.*

to get regularly scheduled screenings and talk with your healthcare team if you have any concerns.

No formal patient education or pre-test counseling was provided following risk assessment completion. However, patients were given study staff contact information for follow-up questions. Those who were not eligible but expressed interest in learning more were sent an email with informational links. Eligible patients who opted-in to the study and provided their contact information for next steps were called by study staff, who answered questions and explained the testing process prior to ordering genetic testing.

Return of Results

There were four possible report options for participants who completed the Color genetic test: (1) positive (pathogenic and/or likely pathogenic), (2) positive with VUS(s), (3) negative with VUS(s) (no pathogenic or likely pathogenic genes identified, but at least one VUS was identified), or (4) negative (no pathogenic, likely pathogenic, or VUS identified).

Results were automatically sent from Color Health to the healthcare system once available and subsequently to patients after a delay to facilitate provider or genetic counselor review and contact in the case of an identified pathogenic variant (PV). As such, patients received results in a number of ways. Study staff manually released negative results through the Color portal before the entire delay period lapsed to minimize waiting time. By design, Color reached out prior to releasing PV results to schedule a genetic counseling appointment by phone. The results were reviewed during that appointment. If the patient did not schedule it after a significant delay, the results were released automatically. Individuals with a negative result could request genetic counseling. In some cases, patients may have been first told by their PCP prior to Color outreach or saw the report upload to their EHR. After the delay lapsed, all results were directly available for patient viewing. In addition to Color outreach, Billings also referred patients with PVs to clinic genetic counselors. Regardless of result, the Color Testing Report, along with any genetic counseling notes, was uploaded to the patients' medical record.

Data Sources

Data is pulled from multiple sources. Data reporting on clinic characteristics or demographics over a 12-month period were pulled from the clinic EHR and do not necessarily reflect the participants in the EDGE study. Data discussing participant demographics are only among those who completed the risk assessment and are pulled from the risk assessment responses.

Actionable and Non-actionable Descriptions Used to Inform Analysis

Actionable variants were those that had clear risk reduction recommendations in the National Comprehensive Cancer Network (NCCN) guidelines. Certain pathogenic or likely pathogenic variants (PVs) were classified as non-actionable, including heterozygous PVs in

autosomal recessive susceptibility genes (i.e. MUTYH) or low penetrant alleles without clear clinical recommendations from the National Comprehensive Cancer Network (NCCN) or other professional organizations.

De-duplication

Patients who visited multiple DPE clinics were de-duplicated prior to study outreach. Patients who visited multiple POC clinics or a combination of POC and DPE clinics were prompted to opt-out of the risk assessment when offered. Remaining incidences were de-duplicated after the fact if sufficient screener information (combination of name, email, phone number) was provided to do so.

Analysis of Aggregate Data at the Site Level

For aggregate outcomes using site-level data only, we performed a series of bivariate logistic regression models with scale adjustment for overdispersion and estimated unadjusted odds ratios (OR) with 95% confidence intervals (CI) and associated p-values testing for differences in odds between study arms comparing POC vs. DPE as the reference category. The dependent variables for these models were the proportion approached, the proportion with familial risk screening completed, the proportion eligible for genetic testing/at risk, the proportion who ordered a test kit, and the proportion whose genetic testing was completed, with the number of patients with a visit during a 12 month study period serving as the denominator. Multivariable logistic regression was used to estimate and test the relative odds between the study arms for these outcomes. Out of *a priori* interest, we controlled for healthcare system (Billings or Multicare) and clinic size (Large or Small). Clinics with patient populations of 8000 per year or more were classified as large clinics and those with populations less than 8000 per year or less were classified as small.

Analysis of Patient-Level Data

We analyzed the patient-level data for those who met the eligibility criteria for genetic testing in both unadjusted and adjusted logistic regression models examining predictors of genetic testing uptake. Apart from study arm, each salient covariate was examined in a simple logistic regression model for potential association with genetic testing and accounting for clustering at the site level using generalized estimating equations (GEE). These covariates included age, sex, personal history of cancer, family history of cancer, healthcare system, and clinic size. We generated unadjusted odds ratios, 95% confidence intervals, and p-values for these analyses. A multivariable logistic regression analysis was then conducted, which included study arm along with all of the covariates. We computed adjusted odds ratios, 95% confidence intervals, and p-values from these models.

In addition, out of *a priori* interest based on the overall study design, we performed stratified multivariable logistic regression by healthcare system and by clinic size. These analyses were augmented by formally testing for heterogeneity of the intervention effect by including interaction terms in separate models for study arm with: a) healthcare system; and b) clinic size.

Finally, using data at the patient-level, we compared the study arms with respect to the presence of an actionable genetic variant overall (any) and by individual variant types in cross-tabulations. We used the overall presence or absence as the dependent variable also in multivariable logistic regression analyses controlling for potential confounding variables that

© 2025 Swisher EM et al. *JAMA Network Open.*

included age, sex, personal history of cancer, family history of cancer, healthcare system, and clinic size. We generated odds ratios, 95% confidence intervals, and p-values from this model.

© 2025 Swisher EM et al. *JAMA Network Open.*
