# Supplementary material for: Strategies to Assess Risk for Hereditary Cancer in Primary Care Clinics: A Cluster Randomized Clinical Trial
Source: JAMA Netw Open. 2025 Mar 7;8(3):e250185. doi: 10.1001/jamanetworkopen.2025.0185 (PMC11889468; doi:10.1001/jamanetworkopen.2025.0185)
Supplement: Supplement 3. — Data Sharing Statement [file jamanetwopen-e250185-s003.pdf]

Data Sharing Statement

Swisher. Strategies to Assess Risk for Hereditary Cancer in Primary Care Clinics. *JAMA Netw Open*. Published March 07, 2025. doi:10.1001/jamanetworkopen.2025.0185

Data
Additional Information: Clinicaltrials.gov NCT04746794
Data available: No

Additional Information
Explanation for why data not available: The data presented in this study are available on request from the corresponding author, ES. Individual data is not available due to privacy restrictions.
